# Role of an ER stress response element in regulating the bidirectional promoter of the mouse *CRELD2 *- *ALG12 *gene pair

**DOI:** 10.1186/1471-2164-11-664

**Published:** 2010-11-25

**Authors:** Kentaro Oh-hashi, Hisashi Koga, Shun Ikeda, Kiyo Shimada, Yoko Hirata, Kazutoshi Kiuchi

**Affiliations:** 1 Department of Biomolecular Science, Faculty of Engineering, Gifu University, 1-1 Yanagido, Gifu 501-1193, Japan; 2 Kazusa DNA Research Institute, 2-6-7 Kazusa-Kamatari, Kisarazu, Chiba 292-0818, Japan

## Abstract

**Background:**

Recently, we identified *cysteine-rich with EGF-like domains 2 *(*CRELD2*) as a novel endoplasmic reticulum (ER) stress-inducible gene and characterized its transcriptional regulation by ATF6 under ER stress conditions. Interestingly, the *CRELD2 *and *asparagine-linked glycosylation 12 homolog *(*ALG12*) genes are arranged as a bidirectional (head-to-head) gene pair and are separated by less than 400 bp. In this study, we characterized the transcriptional regulation of the mouse *CRELD2 *and *ALG12 *genes that is mediated by a common bidirectional promoter.

**Results:**

This short intergenic region contains an ER stress response element (ERSE) sequence and is well conserved among the human, rat and mouse genomes. Microarray analysis revealed that *CRELD2 *and *ALG12 *mRNAs were induced in Neuro2a cells by treatment with thapsigargin (Tg), an ER stress inducer, in a time-dependent manner. Other ER stress inducers, tunicamycin and brefeldin A, also increased the expression of these two mRNAs in Neuro2a cells. We then tested for the possible involvement of the ERSE motif and other regulatory sites of the intergenic region in the transcriptional regulation of the mouse *CRELD2 *and *ALG12 *genes by using variants of the bidirectional reporter construct. With regards to the promoter activities of the *CRELD2*-*ALG12 *gene pair, the entire intergenic region hardly responded to Tg, whereas the *CRELD2 *promoter constructs of the proximal region containing the ERSE motif showed a marked responsiveness to Tg. The same ERSE motif of *ALG12 *gene in the opposite direction was less responsive to Tg. The direction and the distance of this motif from each transcriptional start site, however, has no impact on the responsiveness of either gene to Tg treatment. Additionally, we found three putative sequences in the intergenic region that antagonize the ERSE-mediated transcriptional activation.

**Conclusions:**

These results show that the mouse *CRELD2 *and *ALG12 *genes are arranged as a unique bidirectional gene pair and that they may be regulated by the combined interactions between ATF6 and multiple other transcriptional factors. Our studies provide new insights into the complex transcriptional regulation of bidirectional gene pairs under pathophysiological conditions.

## Background

Among eukaryotes, analyses of the human and mouse genomes revealed that more than 10% of the genes are arranged as bidirectional gene pairs that are separated by less than only 1 kb of genomic DNA [1-3]. Some of these gene pairs could have evolved from a common ancestral gene during its duplication. Other gene pairs, however, do not have any genetic relationship between each other, and they are thought to play different biological functions within cells. It has been reported that the human *PACPG*-*PARK2 *gene pair [4], the human *PREPL*-*C2ORF34 *gene pair [5], the mouse surfeit *Surf1*-*Surf2 *gene pair [6] and the mouse *Sars2*-*Mrps12 *gene pair [7] are co-regulated by distinctive transcriptional factors such as NRF-2, YY-1 or NF-Y. The transcriptional regulation of many other eukaryotic bidirectional gene pairs, however, remains to be determined.

Recently, we identified *CRELD2 *as a novel ER stress-inducible gene by a microarray analysis of Tg-sensitive genes in Neuro2a cells and characterized the 5'-upstream promoter region of the mouse *CRELD2 *gene [8]. Some pathophysiological conditions are reported to disrupt ER functions (e.g., the folding and modifying of newly synthesized transmembrane and secretory proteins) due to an accumulation of unfolded proteins [9,10]. The accumulation of unfolded proteins activates the expression of various genes through three resident ER stress sensors, PERK [11], IRE [12] and ATF6 [13]. The activation of these genes results in various outcomes (e.g., the refolding and/or degradation of accumulated proteins in the ER, and the activation of apoptotic signaling cascades). One of these ER stress sensors, ATF6, directly regulates the transcription of the *CRELD2 *gene via the ERSE motif, an ATF6 consensus sequence, located in its promoter [8]. The nucleotide sequence around the ERSE in the *CRELD2 *promoter is highly conserved within the mouse, rat and human genes. Interestingly, further genomic analyses reveal that the *ALG12 *gene, one of the mannosyltransferase genes [14], is adjacent to the *CRELD2 *gene in a head-to-head configuration on the chromosome in some species. In this study, we first investigated the transcriptional regulation of the bidirectional *CRELD2*-*ALG12 *gene pair as ER stress-inducible genes. We especially focused on evaluating the role of the ERSE motif, which is located within the 360-bp intergenic region, in regulating the expression of both genes under ER-stress conditions.

## Results

### ER stress induced the expression of both *CRELD2 *and *ALG12 *mRNAs in Neuro2a cells

**Figure 1:**
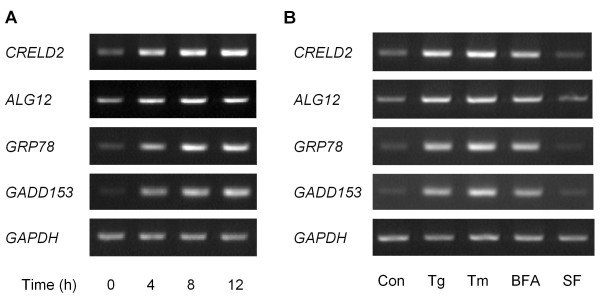
**ER stresses induced *CRELD2 *and *ALG12 *mRNA in Neuro2a cells**. A) Neuro2a cells were treated with 0.1 μM Tg for the indicated time. B) Neuro2a cells were treated with Tg (0.1 μM), Tm (5 μg/ml), BFA (5 μg/ml), serum-free (SF) or vehicle for 6 h. Total RNA isolated from each sample was subjected to RT-PCR as described in methods.

Microarray analyses revealed that both *CRELD2 *and *ALG12 *mRNAs are induced in Tg-treated cells as well as *GADD153 *[15], *Tib3 *[16] and *Herpud1 *[17] mRNA, which are known ER stress-inducible genes (Table 1). To verify the Tg-induced expression of *CRELD2 *and *ALG12 *mRNAs in detail, Neuro2a cells were exposed to 0.1 μM Tg for 4, 8, or 12 h, and the expression of *CRELD2*, *ALG12*, *GRP78 *and *GADD153 *mRNAs were measured by RT-PCR. As shown in Figure 1A, *CRELD2 *and *ALG12 *mRNAs, as well as *GRP78 *[18] and *GADD153 *mRNAs, were up-regulated from 4 to 12 h after Tg-treatment. Next we examined the effects of other ER stress-inducing reagents (Tm and BFA), as well as serum withdrawal, on *CRELD2 *and *ALG12 *mRNA expression in Neuro2a cells. Like Tg treatment, those with Tm and BFA, but not serum withdrawal, induced *CRELD2*, *ALG12*, *GRP78 *and *GADD153 *mRNA expression similarly (Figure 1B).

**Table 1:** Tg-inducible genes in Neuro2a cells

Genes	Name	**RefSeq No**.	Fold induction	
			
			4 h	8 h
*ddit3 (GADD153)*	DNA-damage inducible transcript 3	NM_007837	6.1	9.8
*trib3*	tribbles homolog 3	NM_175093	3.9	7.2
*herpud1*	homocysteine-inducible, endoplasmic reticulum stress-inducible, ubiquitin-like domain member 1	NM_022331	4.4	6.0
***creld2***	cysteine-rich with EGF-like domains 2	NM_029720	2.9	4.3
***alg12***	asparagine-linked glycosylation 12 homolog	NM_145477	3.6	3.7

Neuro2a cells were treated with or without 0.1 μM Tg for 4 or 8 h. Total RNA was isolated from each cells and subjected to identify Tg-inducible genes using microarray analysis as described in methods. Representative genes are shown as ratio (Tg-treated cells/control cells).

### Comparison of the intergenic sequences of the *CRELD2*-*ALG12 *gene pair within the mouse, rat and human genes

**Figure 2:**
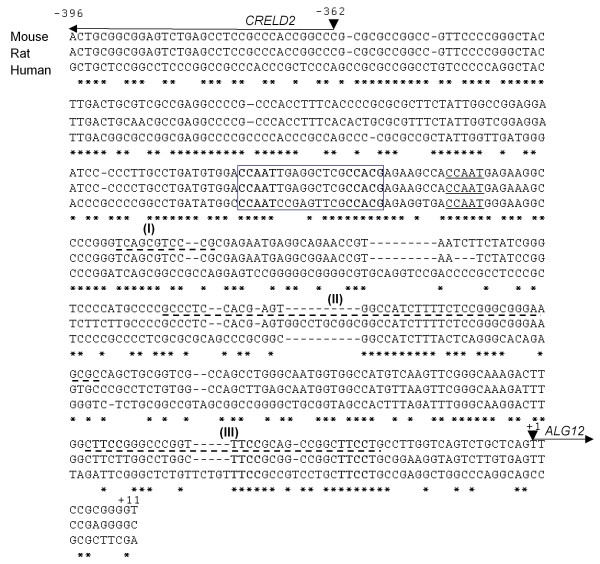
**Nucleotide sequences of the mouse, rat and human *CRELD2*-*ALG12 *gene pairs**. Nucleotide sequences conserved among the mouse, rat and human *CRELD2*-*ALG12 *gene pairs are shown with asterisks. A conserved ERSE motif and an adjacent NF-Y binding site are shown in a box and underline, respectively. The transcriptional direction and putative transcriptional start sites of the mouse *CRELD2 *and *ALG12 *genes are shown with arrows and arrowheads, respectively. Three suppressive regions identified in this study are shown with broken lines (sites I, II and III). Putative binding sequences of the Ets family (TTCC) in site III are shown with bold types.

**Figure 3:**
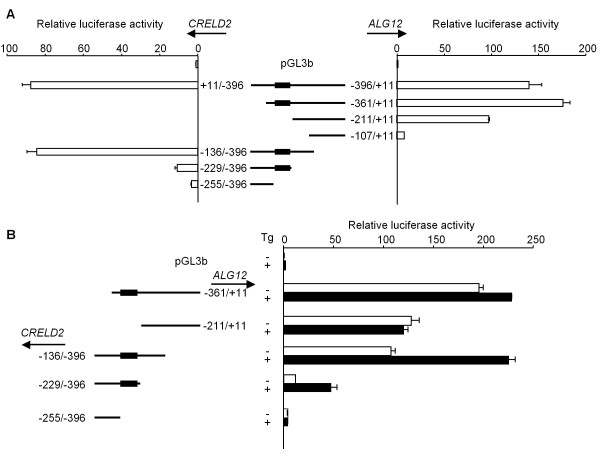
**Promoter activity of the mouse *CRELD2*-*ALG12 *gene pair in Neuro2a cells**. Neuro2a cells were transfected with various luciferase reporter constructs containing successive 5'-deletion mutations of the mouse *CRELD2 *and *ALG12 *promoter sequences. A) Thirty-six hours after transfection, luciferase activity was measured. B) Twenty-four hours after transfection, the cells were incubated with or without Tg (0.1 μM) for 10 h, and luciferase activity was measured. The promoter activity in the absence (-) or presence (+) of Tg is shown as open and closed columns, respectively. Values represent means ± SD from 3 independent cultures and are expressed relative to the basal activity of the pGL3-Basic vector.

Next we analyzed the intergenic sequences of the *CRELD2*-*ALG12 *gene pair within the mouse, rat and human genes. As shown in Figure 2, the nucleotide sequence of the mouse gene pair is highly homologous to that of the rat gene pair. The proximal promoter regions of the human and mouse *CRELD2 *genes, especially around the ERSE motif, are also well conserved. We then measured the basal promoter activities of the mouse *CRELD2*-*ALG12 *gene pair by using luciferase reporter constructs inserted with either the entire intergenic region (-396 ~ +11) or the intergenic region containing various deletion mutations in either direction. As shown in Figure 3A, reporter constructs containing the entire intergenic region in either direction (+11/-396; *CRELD2 *promoter and -396/+11; *ALG12 *promoter) showed the higher basal promoter activity. The activity of *ALG12 *promoter (-211/+11) is still high in the absence of the ERSE motif, however a further deletion from position -211 to -108 in this promoter remarkably decreased its basal activity in Neuro2a cells. Furthermore, a deletion from position -136 to -228 in the *CRELD2 *promoter dramatically decreased *CRELD2 *promoter activity even though the ERSE motif is present. The deletion of a region around the ERSE motif (-229 ~ -254) further decreased the promoter activity.

### The role of the ERSE motif in *CRELD2 *and *ALG12 *promoter activities under ER stress condition

**Figure 4:**
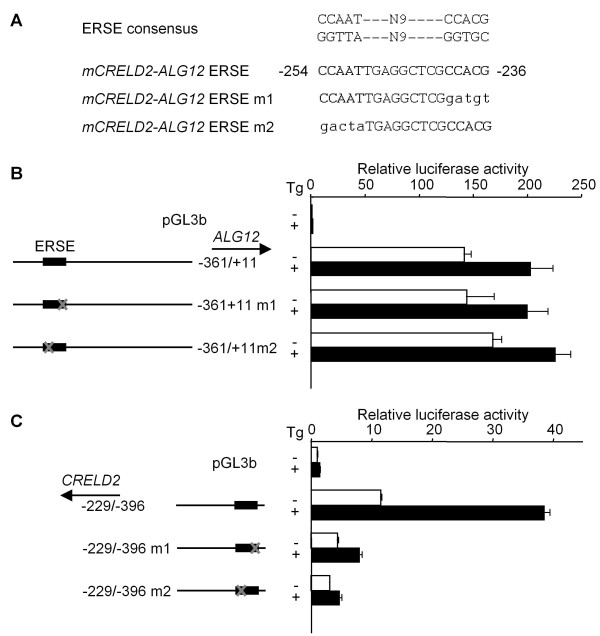
**Role of the ERSE motif in mouse *CRELD2 *and *ALG12 *promoter activities in Neuro2a cells**. A) The nucleotide sequence of a common ERSE motif in the mouse *CRELD2-ALG12 *gene is shown. NF-Y and ATF6 binding sequences in the ERSE motif and their mutated sequences are shown with bold types and small letters, respectively. Neuro2a cells were transfected with the indicated *CRELD2 *or *ALG12 *reporter constructs. Twenty-four hours after transfection, the cells were incubated with or without Tg (0.1 μM) for 10 h (B and C). After incubation, the cells were lysed and luciferase activity was measured as described in methods. The activities obtained from control and Tg-treated cells are shown as open and closed columns, respectively. Values represent means ± SD from 3 - 4 independent cultures and are expressed relative to the basal activity of the pGL3-Basic.

**Figure 5:**
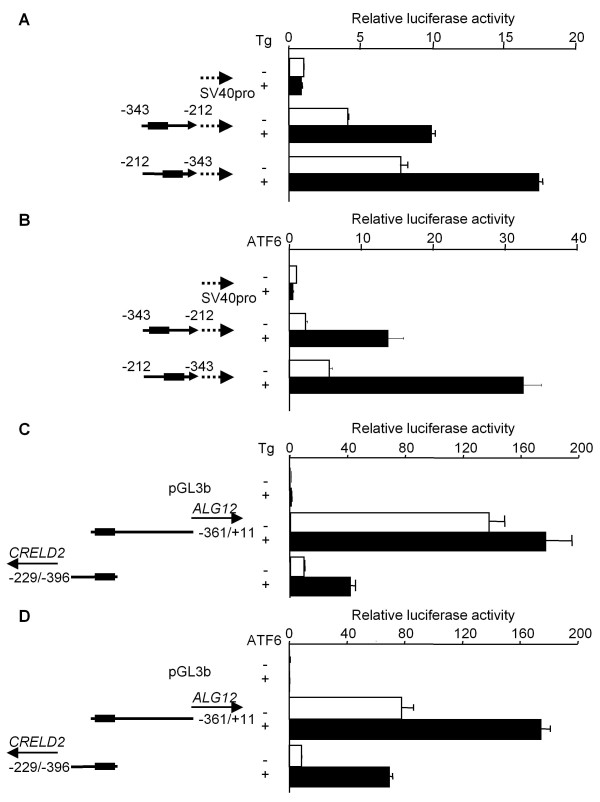
**Role of nucleotide sequence around the ERSE motif in mouse *CRELD2 *and *ALG12 *promoters in regulating the transcriptional activity of both genes**. Neuro2a cells were transfected with the indicated *CRELD2 *or *ALG12 *reporter constructs. Twenty-four hours after transfection, the cells were incubated with or without Tg (0.1 μM) for 10 h (A and C). The expression vector for ATF6 or an empty vector (mock) was co-transfected with each reporter construct into Neuro2a cells and cultured for 36 h (B and D). After incubation, the cells were lysed and luciferase activity was measured as described in methods. Control and Tg-treated or ATF6-stimulated activities are shown as open and closed columns, respectively. Values represent means ± SD from 3 - 4 independent cultures and are expressed relative to the basal activity of the pGL3-Promoter (A and B) and pGL3-Basic (C and D).

As shown in Figure 3B, the mouse *CRELD2 *promoter containing the proximal region (-229 ~ -396), but no deletion mutation construct of mouse *ALG12 *promoter, was significantly activated by Tg-treatment. Consistent with our previous report, the *CRELD2 *promoter construct containing the longer intergenic region (-136 ~ -396) showed higher basal promoter activity but a lower responsiveness to Tg compared to the above-mentioned construct (-229/-396) (Figure 3B). The *CRELD2 *promoter without the ERSE motif (-255/-396) had an even further diminished basal promoter activity and Tg- responsiveness. Next, we determined the effect of various mutations within the ERSE motif on the activity of the mouse *ALG12 *promoter. Unlike the *CRELD2 *promoter (-229/-396) and its point mutation constructs (-229/-396 m1 or m2; a mutation at the ATF6 or NF-Y binding site [8,13,18-20], respectively), the mutations in the *ALG12 *promoter (-361/+11 m1 and m2) did not affect the basal promoter activity and the responsiveness to Tg (Figure 4). Then, we evaluated the effect of the ERSE motif's direction on the responsiveness of the mouse *CRELD2*-*ALG12 *gene pair to Tg by using a pGL3 vector containing the SV40-promoter. The reporter constructs containing a partial intergenic region of the gene pair (-343 ~ -212) in either direction responded to Tg and ATF6-overexpression similarly (Figure 5A and 5B). Interestingly, Tg-treatment and ATF6-overexpression stimulated the luciferase activity of the *CRELD2 *promoter (-229/-396) construct more effectively than the *ALG12 *promoter (-361/+11) construct (Figure 5C and 5D).

**Figure 6:**
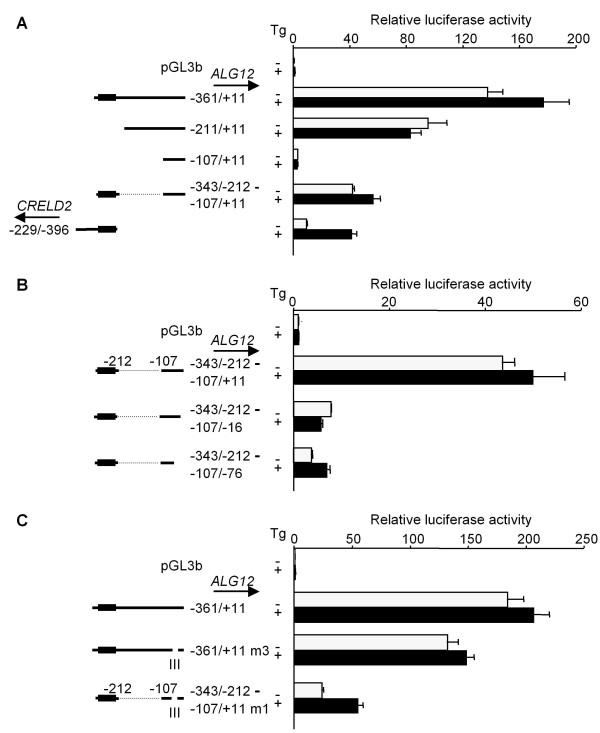
**Role of the proximal region of the mouse *ALG12 *promoter in regulating the transcriptional activity of the gene**. Neuro2a cells were transfected with the indicated *CRELD2 *or *ALG12 *reporter constructs. Twenty-four hours after transfection, the cells were incubated with or without Tg (0.1 μM) for 10 h, and luciferase activity was measured. A deleted site in the most proximal region of the *ALG12 *promoter is shown as site III. Control and Tg-treated activities are shown as open and closed columns, respectively. Values represent means ± SD from 3 independent cultures and are expressed relative to the basal activity of the pGL3-Basic vector (A, B and C).

**Figure 7:**
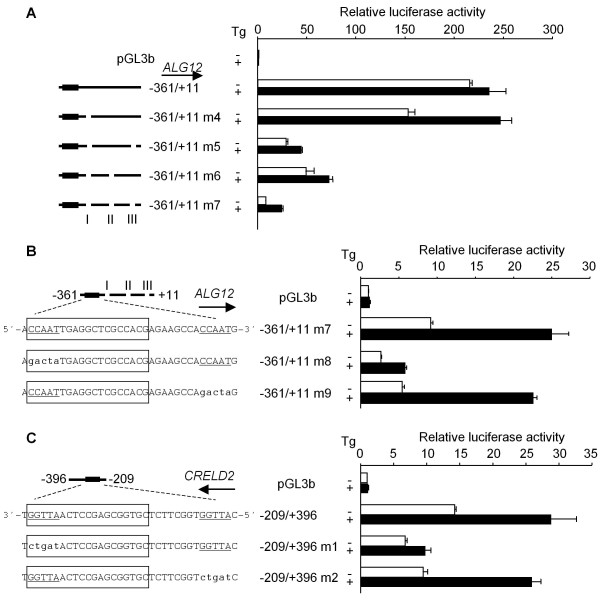
**Characterization of the suppressive sites affecting the activity of the mouse *CRELD2 *and *ALG12 *promoters**. Neuro2a cells were transfected with the indicated *CRELD2 *or *ALG12 *reporter constructs. Twenty-four hours after transfection, the cells were incubated with or without Tg (0.1 μM) for 10 h, and the luciferase activity was measured (A, B and C). Each deleted suppressive site in the *CRELD2 *and *ALG12 *promoters is shown as site I, II or III. The ERSE motif and two NF-Y binding sites in the mouse *CRELD2 *and *ALG12 *reporters are shown with a box and underlines, respectively. The nucleotide sequence of the each mutated NF-Y binding site is shown with small letters (B and C). Control and Tg-treated activities are shown as open and closed columns, respectively. Values represent means ± SD from 3 independent cultures and are expressed relative to the basal activity of the pGL3-Basic vector.

**Figure 8:**
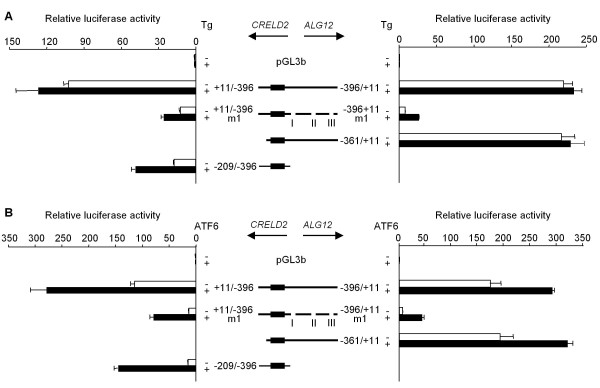
**Characterization of the promoter activity of the full intergenic region of the mouse *CRELD2*-*ALG12 *gene pair**. A) Neuro2a cells were transfected with the indicated *CRELD2 *or *ALG12 *reporter constructs. Twenty-four hours after transfection, the cells were incubated with or without Tg (0.1 μM) for 10 h, and the luciferase activity was measured. B) The expression vector for ATF6 or an empty vector (mock) was co-transfected with each reporter construct into Neuro2a cells. Thirty-six hours after transfection, luciferase activity for each construct was measured. Control and Tg- or ATF6-stimulated activities are shown as open and closed columns, respectively. Values represent means ± SD from 3 independent cultures and are expressed relative to the basal activity of the pGL3-Basic vector.

To study the unresponsiveness of the *ALG12 *promoter (-361/+11) to Tg, we prepared another reporter construct in which the middle intergenic region of the *ALG12 *promoter (-211 ~ -108) that contributes to the basal promoter activity is deleted (Figure 3A). This construct (-343/-212 & -107/+11), however, did not respond to Tg (Figure 6A). Serial deletions of the 3'-end of the *ALG12 *promoter lacking the middle intergenic region revealed that there is a suppressive site from position -75 to -16 in the *ALG12 *promoter (Figure 6B). Deletion around three putative Ets family binding sites (TTCC) [21,22] from position -52 to -20 (Figure 2, site III) in the *ALG12 *promoter (-343/-212 & -107/+11 m1) also restored responsiveness to Tg. Yet, this same site III deletion in the *ALG12 *promoter containing the middle intergenic region (-361/+11 m3) showed unresponsiveness to Tg (Figure 6C). To determine if there are other suppressive sites in this intergenic region, we prepared various deletion mutation constructs of the *ALG12 *promoter and evaluated their responsiveness to Tg. As shown in Figure 7A, we identified two additional suppressive sites (I and II in Figure 2). We also found that the deletion of all three sites (-361/+11 m7; *ALG12 *promoter) was required in order to restore the responsiveness to Tg. A mutation in either the NF-Y binding site (CCAAT) of the ERSE motif (-361/+11 m8) or a site 8-bp downstream of the ERSE motif (-361/+11 m9) in the *ALG12 *promoter showed that each NF-Y binding site partially participated in its basal promoter activity. Only the site in the ERSE motif in the *ALG12 *promoter (-361/+11 m8), however, are crucial to the responsiveness to Tg as well as the *CRELD2 *promoter (-209/+396 m1) (Figure 7B and 7C). Finally, we measured the promoter activity of the entire intergenic region of the *CRELD2*-*ALG12 *gene pair in the both direction (-396/+11; *CRELD2 *and *ALG12 *promoters) after Tg treatment or ATF6 cotransfection. Both promoter constructs only slightly responded to Tg, but the deletion of the three suppressive regions (+11/-396 m1; *CRELD2 *promoter and -396/+11 m1; *ALG12 *promoter) restored responsiveness to Tg. Furthermore, the basal promoter activities of these mutant constructs markedly decreased (Figure 8A). ATF6-overexpression enhanced the promoter activity of all of the above-mentioned constructs. The Tg-responsive reporter constructs (+11/-396 m1 and -209/-396; *CRELD2 *promoters and -396/+11 m1; *ALG12 *promoter) also showed a further increase in their promoter activities by ATF6-overexpression (Figure 8B).

## Discussion

Recently, we identified *CRELD2 *as a novel ER-stress inducible gene and characterized its ATF6-dependent transcriptional regulation using constructs containing the proximal region of the mouse *CRELD2 *promoter [8]. Genomic analyses reveal that the *ALG12 *gene [14] is adjacent to the *CRELD2 *gene in a head-to-head configuration on the chromosome in some species. *CRELD2 *and *ALG12 *genes are a bidirectional gene pair arranged less than 400 bp apart. The nucleotide sequences of this intergenic region are moderately conserved among the mouse, rat and human genes. Furthermore, those regions around an ERSE motif in the *CRELD2*-*ALG12 *gene pair are highly conserved (Figure 2). In this study, we demonstrate that the expression of *CRELD2 *and *ALG12 *mRNAs, and *GRP78 *and *GADD153 *mRNAs, which are well known ER stress-inducible genes, was induced by three distinct ER stress inducers (Figure 1). In regards to the promoter activity of the mouse *CRELD2*-*ALG12 *gene pair, only the *CRELD2 *promoter containing just the proximal region (-229/-396 and -209/-396; *CRELD2 *promoter) significantly responded to Tg. Additionally, the *CRELD2 *promoter containing the full intergenic region (+11/-396; *CRELD2 *promoter) decreased in responsiveness to Tg, whereas its basal promoter activity markedly increased. In contrast, the *ALG12 *promoters only slightly responded to Tg even though some of the reporters contained the ERSE motif, which is 300-bp apart from the transcription start site of the mouse *ALG12 *gene. The direction of the ERSE motif and its distance from each of the transcription start sites for the mouse *CRELD2 *or *ALG12 *genes, however, appear to have no influence in these findings. Therefore, it seems that the full intergenic region contains one or more unknown suppressive sites that interfere with the ERSE-mediating enhancement of the *ALG12 *and *CRELD2 *promoter activities.

Reporter constructs used in this study contain 5'-untranslated regions (5'-UTR) of *CRELD2 *and/or *ALG12 *gene. Especially, reporter constructs containing the entire intergenic region of *CRELD2 *- *ALG12 *gene pair contain the UTR regions at both ends. However, the deletion of three suppressive sites (sites I, II and III) in each construct recovered the responsiveness to Tg (-343/-212 - -107/+11 m1 in Figure 6C and -361/+11 m7, +11/-396 m1 and -396/+11 m1 in Figure 7A and 8A). Therefore, it seems likely that each 5'-UTR hardly influenced the corresponding promoter activity of the *CRELD2 *and *ALG12 *promoter constructs in our assay system. *CRELD2 *(NM_029720.2) and *ALG12 *(NM_145477.1) genes possess 5'-UTR (66 and 55 bp) and 3'-UTR (245 and 584 bp) respectively though their effects on transcription are not elucidated yet. Further characterization of these regions would reveal regulations of *CRELD2 *and *ALG12 *mRNA expression.

**Figure 9:**
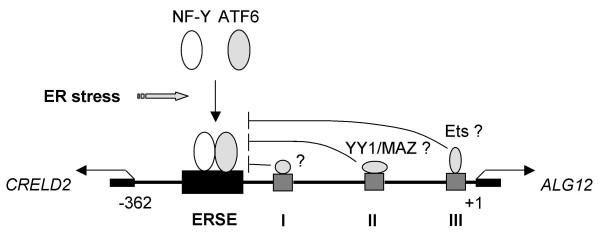
**Proposed mechanisms regulating promoter activities in the intergenic region of *CRELD2 *and *ALG12 *gene pair**. Under ER stress conditions, ATF6 and NF-Y translocate into nucleus, form complex and specifically bind the ERSE motif to induce *CRELD2 *and *ALG12 *promoter activities. Three sites (I, II and III) in the intergenic region identified in this study are important for their basal promoter activities. However, factors binding to the site I, II and III could antagonize the ERSE-mediated up-regulation of both promoter activities cooperatively. According to the database of transcription regulatory motifs, the site II contains putative YY1- and MAZ-binding motifs and the site III contains an Ets family-binding motif, respectively.

Using various deletion mutation constructs, we showed that three suppressive sites in the *CRELD2*-*ALG12 *gene pair play a crucial role in interfering with Tg responsiveness. Interestingly, the deletion of all three of these suppressive sites was required in order to restore the responsiveness to Tg (Figure 2, 7 and 8). These results imply that these suppressive sites are not only important in maintaining basal promoter activity, but that they synergistically counteract the ERSE-mediated transcriptional activity (Figure 9). Among these sites, the most proximal to the *ALG12 *promoter contains a conserved response element that Ets-family transcriptional factors recognize [21]. Ets transcription factors consist of approximately 30 family members and share a highly conserved DNA-binding domain. It has been reported that these factors are involved in regulating a variety of biological processes including development, differentiation and inflammation [21,22]. In the site II, there are putative YY1- (GCCATC) [23] and MAZ- (CCCCGCCCT) [24] binding sites judged from some databases such as SwissRegulon (a database of genome-wide annotations of regulatory sites), but the precise roles remain to be determined. On the contrary, we are unable to find any unique sequences in the sites I. Further studies characterizing each of these suppressive sites (site I, II, and III) are required in order to understand the complex transcriptional regulation of the *CRELD2*-*ALG12 *gene pair. Jones PL *et al. *reported that murine manganese superoxide dismutase gene is regulated through a complex intronic enhancer involving C/EBP-β and NF-κB [25]. Donati G *et al. *demonstrated that ER stress triggers dynamic modification of chromatin components and transcriptional factors under ER stress [20]. Therefore, we should focus on other aspects such as local chromatin remodeling and histone modifications (e.g., phosphorylation, acetylation and methylation of histones) within the *CRELD2 *and *ALG12 *genes in addition to the 5'-flanking sequences in this intergenic region. Furthermore, other approaches should be employed to elucidate the discrepancy between the expression levels of both intrinsic mRNAs and the promoter activities of their full intergenic region under ER stress conditions.

Among the bidirectional gene pairs characterized in mammalian cells, *Surf1*-*Surf2 *[6]*, Reql4*-*Lrrc14 *[26], *PDCD10*-S*ERPINI1 *[27] and *Thox*-*DUOXA *[28] gene pairs seem to share their intergenic region equally because mutations in the transcription factor binding sites decline those promoter activities equally. In contrast, the transcriptional regulations of *C2ORF34*-*PREPL *[5], *Sarsm*-*Mrps1*2 [7] and *HAND2*-*DEIN *[29] are asymmetric. According to the present study, the transcriptional regulatory pattern of the mouse *CRELD2*-*ALG12 *gene pair belongs to the latter group. Analyses of these bidirectional gene pair sharing a common intergenic region have mostly consisted of characterization without any stimuli. Recently, Zanotto E *et al. *reported that the *Sarsm*-*Mrps12 *promoter activity is modulated by mitochondrial stresses, especially mitochondrial reactive oxygen species, in a complex manner [30]. At this time, however, the significance and relevance of many bidirectional gene pairs under pathophysiological conditions are not well understood.

The mammalian *ALG12 *gene is the ortholog of the yeast gene that encodes the dolichyl-P-Man:Man_7_GlcNAc_2_-PP-dolichyl α6-mannosyltransferase, and its mutation causes a congenital disorder affecting glycosylation in the ER [14,31,32]. Clinically, a child suffering from a point mutation in the *ALG12 *gene has been reported to show severe symptoms such as psychomotor retardation, hypotonia, growth retardation, dysmorphic features and anoxia [31]. Sequential protein glycosylation in the ER is important in maintaining the quality control of glycoproteins through folding and ER-associated protein degradation. Moreover, its defects could also interfere with the intracellular trafficking and secretion of glycoproteins. Therefore, suitable regulation of the *ALG12 *gene should be required in order to maintain ER homeostasis.

As the CRELD proteins have multiple EGF-like domains, they are considered to be cell adhesion molecules [33-35]. It has been reported that missense mutations in the *CRELD1 *gene increases an individual's susceptibility to atrioventricular septal defects [33], but the physiological roles of these family members remain poorly understood. In contrast to CRELD1, CRELD2 lacks a transmembrane domain in the C-terminal region. Ortiz *et al*. reported that the overexpression of CRELD2 impairs the membrane transport of acetylcholine receptor α4/β2 in *Xenopus laevis *oocytes [36]. We recently demonstrated that the *CRELD2 *gene is one of the downstream targets of ATF6 and that its product is predominantly localized in the ER-Golgi apparatus [8]. Interestingly, the mouse model for multiple epiphyseal dysplasia, which specifically expresses a mutation in matrilin-3, was reported to induce *CRELD2 *mRNA expression and other ER-stress inducible genes as the symptoms progressed [37]. According to these reports, CRELD2 seems to be involved in the folding, processing and transport of some proteins under pathophysiological conditions, though the precise role of CRELD2 remains to be determined. Furthermore, we believe that the sharing of the ERSE motif in the *CRELD2*-*ALG12 *gene pair may be advantageous in regulating ER homeostasis under various ER-stress conditions, even though it is unlikely that the CRELD2 and ALG12 proteins function by directly interacting with each other.

## Conclusion

In this study, we first demonstrate that both the *CRELD2 *and *ALG12 *genes, which form a bidirectional gene pair, are potent ER stress-inducible genes. Our present results indicate that the *CRELD2*-*ALG12 *gene pair could be asymmetrically regulated by multiple transcriptional factors in addition to ATF6. Because the *CRELD2*-*ALG12 *gene pair contains an evolutionally conserved ERSE motif, the cooperative induction of these genes may play important roles in confronting ER stresses and in appropriately regulating ER homeostasis and cell fates, together with other ER stress-inducible genes (e.g., *GRP78 *and *GADD153*). Therefore, further characterization of the *CRELD2*-*ALG12 *gene pair may provide new insights into the complex transcriptional regulation of ER stress-inducible genes as well as into the onset and progression of various ER stress-associated diseases.

## Methods

### Cell culture and treatment

Neuro2a cells were maintained in Dulbecco's Modified Eagles minimum essential Medium containing 8% fetal bovine serum. Transfection of each construct used in this study was performed using Lipofectamine-Plus reagent (Invitrogen) according to the manufacturer's instructions. For stimulation, Neuro2a cells were treated with Tg (0.1 μM), Tm (5 μg/ml), BFA (5 μg/ml) or serum-free medium (SF) for the indicated time.

### Construction of plasmids

For preparation of reporter constructs for the mouse *CRELD2 *and *ALG12 *promoters, genomic DNA from Neuro2a cells was extracted, and the mouse *CRELD2 *and *ALG12 *promoters were amplified by polymerase chain reaction (PCR) and cloned into the pGL3-Basic vector (Promega). To evaluate the promoter activity of the intergenic region of the mouse *CRELD2 *and *ALG12 *genes, the position of the putative transcriptional start site of mouse *CRELD2 *or *ALG12 *is defined as -362 and +1, respectively. The promoter region was defined using a database of the NIH full-length cDNA project and RIKEN functional annotation of a full-length mouse cDNA collection (FANTOM). To characterize the enhancer activity of the partial intergenic region containing ERSE (-343 ~ -212), it was inserted into the pGL3-Promoter vector (Promega). We also constructed various other bidirectional reporter construct carrying point and deletion mutations. Mouse ATF6 was amplified by PCR using cDNA from Neuro2a cells and cloned into the pFlag-CMV vector.

### Reverse transcription polymerase chain reaction (RT-PCR)

To estimate the expression level of each gene by RT-PCR, total RNA was extracted from cells lysed with Trizol and converted to cDNA by reverse transcription using random ninemers to prime superscript III RNase^- ^reverse transcriptase (RT) (Invitrogen) as previously described [8]. Specific cDNAs were mixed and amplified with a PCR reaction mixture (Taq PCR kit, Takara). The RT-PCR primers used in this study were as follows: *CRELD2 *sense primer 5'-ACTGAAGAAGGAGCACCCCAAC-3', *CRELD2 *antisense primer 5'-GCACACTCATCCACATCCACACA-3', *ALG12 *sense primer 5'-GTGATTTCTGGACTCTGGAC-3', *ALG12 *antisense primer 5'-GGGGTATGAAGAGAAGGCTGCA-3', *GADD153 *sense primer 5'-GAATAACAGCCGGAACCTGA-3', *GADD153 *antisense primer 5'-GGACGCAGGGTCAAGAGTAG-3', *GRP78 *sense primer 5'-ACCAATGACCAAAACCGCCT-3', *GRP78 *antisense primer 5'-GAGTTTGCTGATAATTGGCTGAAC-3', *GAPDH *sense primer 5'-ACCACAGTCCATGCCATCAC-3', *GAPDH *antisense primer 5'-TCCACCACCCTGTTGCTGTA-3'. The typical reaction cycle conditions were 30 sec at 96°C, 30 sec at 60°C and 30 sec at 72°C. The results represent 18 ~ 33 cycles of amplification, after which cDNAs were separated by electrophoresis on 2.0% agarose gels and visualized using ethidium bromide. Experiments were repeated to confirm reproducibility.

### GeneChip analysis

After Neuro2a cells were incubated in the absence or presence of Tg for the indicated time, total RNA was extracted as described in the above methods. After measuring the quantity and quality of the RNA, biotin-labeled cRNAs were generated from 5 μg of each total RNA using a GeneChip^® ^One-Cycle Target Labeling and Control Reagents package (Affymetrix) according to the manufacturer's protocol. Afterwards, 15 μg of the purified cRNAs were mixed with 3 nM Control Oligo B2, and the hybridization cocktail was denatured at 99°C for 5 min in a heat block, followed by incubation at 45°C for 5 min, and centrifugation for 5 min in order to remove any insoluble material. Hybridization to a mouse DNA array (GeneChip^® ^Mouse Genome 430 2.0 Array) was carried out at 45°C for 16 h using a hybridization oven 640 (Affymetrix). After hybridization, the arrays were washed and stained with the GeneChip^® ^Hybridization Wash and Stain Kit (Affymetrix) using the GeneChip^® ^Fluidics Station 450 (Affymetrix) according to the manufacturer's protocol. The signal intensities were quantified using a GeneArray Scanner 3000 (Affymetrix), and the raw data obtained were converted into MAS (Microarray Suite) files using the GeneChip^® ^Operating Software (GCOS). After normalization, the identification of the temporal expression patterns of genes was performed using the Spotfire^® ^DecisionSite. In this analysis, the mean signal intensity of gene expression in each group included in the study (three samples/group) was used. As a selection criteria to present only the most relevant genes, a cutoff of a 2.0-fold increased/decreased expression and a p < 0.01 were arbitrarily chosen.

### Reporter gene assay

Reporter constructs and the pRL-TK vector, an internal control, were transfected into Neuro2a cells in a 48-well plate. Twenty-four hours after transfection, the cells were treated with Tg (0.1 μM) or vehicle for 10 ~ 12 h. To determine the effects of ATF6 on reporter activity, the ATF6 expression vector or empty vector (mock) was co-transfected with the reporter construct into the cells and cultured for 36 h. After incubation under each condition, the cells were lysed and the luciferase activity in each lysate was measured using a Dual-Luciferase assay system (Promega). Reporter activity in each lysate was normalized to the co-transfected Renilla luciferase activity, and the results are shown as relative luciferase activity.

## Authors' contributions

KO conceived of this study, carried out the molecular genetics studies, participated in the sequence alignment and drafted the manuscripts. HK, SI and KS participated in microarray analysis. YH and KK participated in its design and coordination. All authors read and approved the final manuscript.

## References

[B1] Trinklein ND Aldred SF Hartman SJ Schroeder DI Otillar RP Myers RM An abundance of bidirectional promoters in the human genome Genome Res 2004 14 1 62 66 10.1101/gr.1982804 14707170 PMC314279

[B2] Li YY Yu H Guo ZM Guo TQ Tu K Li YX Systematic analysis of head-to-head gene organization: evolutionary conservation and potential biological relevance PLoS Comput Biol 2006 2 7 e74 10.1371/journal.pcbi.0020074 16839196 PMC1487180

[B3] Adachi N Lieber MR Bidirectional gene organization: a common architectural feature of the human genome Cell 2002 109 7 807 809 10.1016/S0092-8674(02)00758-4 12110178

[B4] Agirre X Román-Gómez J Vázquez I Jiménez-Velasco A Garate L Montiel-Duarte C Artieda P Cordeu L Lahortiga I Calasanz MJ Heiniger A Torres A Minna JD Prósper F Abnormal methylation of the common PARK2 and PACRG promoter is associated with downregulation of gene expression in acute lymphoblastic leukemia and chronic myeloid leukemia Int J Cancer 2006 118 8 1945 1953 10.1002/ijc.21584 16287063

[B5] Huang CC Chang WS Cooperation between NRF-2 and YY-1 transcription factors is essential for triggering the expression of the PREPL-C2ORF34 bidirectional gene pair BMC Mol Biol 2009 10 67 10.1186/1471-2199-10-67 19575798 PMC2713978

[B6] Gaston K Fried M YY1 is involved in the regulation of the bi-directional promoter of the Surf-1 and Surf-2 genes FEBS Lett 1994 347 2-3 289 294 10.1016/0014-5793(94)00567-2 8034020

[B7] Zanotto E Shah ZH Jacobs HT The bidirectional promoter of two genes for the mitochondrial translational apparatus in mouse is regulated by an array of CCAAT boxes interacting with the transcription factor NF-Y Nucleic Acids Res 2007 35 2 664 677 10.1093/nar/gkl1037 17179180 PMC1802594

[B8] Oh-hashi K Koga H Ikeda S Shimada K Hirata Y Kiuchi K CRELD2 is a novel endoplasmic reticulum stress-inducible gene Biochem Biophys Res Commun 2009 387 3 504 510 10.1016/j.bbrc.2009.07.047 19615339

[B9] Kim I Xu W Reed JC Cell death and endoplasmic reticulum stress: disease relevance and therapeutic opportunities Nat Rev Drug Discov 2008 7 12 1013 1030 10.1038/nrd2755 19043451

[B10] Lindholm D Wootz H Korhonen L ER stress and neurodegenerative diseases Cell Death Differ 2006 13 3 385 392 10.1038/sj.cdd.4401778 16397584

[B11] Harding HP Zhang Y Ron D Protein translation and folding are coupled by an endoplasmic-reticulum-resident kinase Nature 1999 397 6716 271 274 10.1038/16729 9930704

[B12] Calfon M Zeng H Urano F Till JH Hubbard SR Harding HP Clark SG Ron D IRE1 couples endoplasmic reticulum load to secretory capacity by processing the XBP-1 mRNA Nature 2002 415 6867 92 96 10.1038/415092a 11780124

[B13] Zhu C Johansen FE Prywes R Interaction of ATF6 and serum response factor Mol Cell Biol 1997 17 9 4957 4966 9271374 10.1128/mcb.17.9.4957 PMC232347

[B14] Burda P Jakob CA Beinhauer J Hegemann JH Aebi M Ordered assembly of the asymmetrically branched lipid-linked oligosaccharide in the endoplasmic reticulum is ensured by the substrate specificity of the individual glycosyltransferases Glycobiology 1999 9 6 617 625 10.1093/glycob/9.6.617 10336995

[B15] Ron D Habener JF CHOP, a novel developmentally regulated nuclear protein that dimerizes with transcription factors C/EBP and LAP and functions as a dominant-negative inhibitor of gene transcription Genes Dev 1992 6 3 439 453 10.1101/gad.6.3.439 1547942

[B16] Ohoka N Yoshii S Hattori T Onozaki K Hayashi H TRB3, a novel ER stress-inducible gene, is induced via ATF4-CHOP pathway and is involved in cell death EMBO J 2005 24 6 1243 1255 10.1038/sj.emboj.7600596 15775988 PMC556400

[B17] Kokame K Agarwala KL Kato H Miyata T Herp, a new ubiquitin-like membrane protein induced by endoplasmic reticulum stress J Biol Chem 2000 275 32846 32853 10.1074/jbc.M002063200 10922362

[B18] Yoshida H Haze K Yanagi H Yura T Mori K Identification of the cis-acting endoplasmic reticulum stress response element responsible for transcriptional induction of mammalian glucose-regulated proteins. Involvement of basic leucine zipper transcription factors J Biol Chem 1998 273 50 33741 33749 10.1074/jbc.273.50.33741 9837962

[B19] Li M Baumeister P Roy B Phan T Foti D Luo S Lee AS ATF6 as a transcription activator of the endoplasmic reticulum stress element: thapsigargin stress-induced changes and synergistic interactions with NF-Y and YY1 Mol Cell Biol 2000 20 14 5096 5106 10.1128/MCB.20.14.5096-5106.2000 10866666 PMC85959

[B20] Donati G Imbriano C Mantovani R Dynamic recruitment of transcription factors and epigenetic changes on the ER stress response gene promoters Nucleic Acids Res 2006 34 10 3116 3127 10.1093/nar/gkl304 16757577 PMC1475745

[B21] Oettgen P Regulation of vascular inflammation and remodeling by ETS factors Circ Res 2006 99 11 1159 1166 10.1161/01.RES.0000251056.85990.db 17122446

[B22] Chung SW Chen YH Perrella MA Role of Ets-2 in the regulation of heme oxygenase-1 by endotoxin J Biol Chem 2005 280 6 4578 4584 10.1074/jbc.M409125200 15590657

[B23] Shi Y Seto E Chang LS Shenk T Transcriptional repression by YY1, a human GLI-Kruppel-related protein, and relief of repression by adenovirus E1A protein Cell 1991 67 2 377 388 10.1016/0092-8674(91)90189-6 1655281

[B24] Bossone SA Asselin C Patel AJ Marcu KB MAZ, a zinc finger protein, binds to c-MYC and C2 gene sequences regulating transcriptional initiation and termination Proc Natl Acad Sci USA 1992 89 16 7452 7456 10.1073/pnas.89.16.7452 1502157 PMC49728

[B25] Jones PL Ping D Boss JM Tumor necrosis factor α and interleukin-1β regulate the murine manganese superoxide dismutase gene through a complex intronic enhancer involving C/EBP-β and NF-κB Mol Cell Biol 1997 17 12 6970 6981 9372929 10.1128/mcb.17.12.6970 PMC232554

[B26] Uwanogho DA Yasin SA Starling B Price J The intergenic region between the Mouse Recql4 and Lrrc14 genes functions as an evolutionary conserved bidirectional promoter Gene 2010 449 1-2 103 117 10.1016/j.gene.2009.08.011 19720120

[B27] Chen PY Chang WS Lai YK Wu CW c-Myc regulates the coordinated transcription of brain disease-related PDCD10-SERPINI1 bidirectional gene pair Mol Cell Neurosci 2009 42 1 23 32 10.1016/j.mcn.2009.05.001 19442737

[B28] Christophe-Hobertus C Christophe D Delimitation and functional characterization of the bidirectional THOX-DUOXA promoter regions in thyrocytes Mol Cell Endocrinol 2010 317 1-2 161 167 10.1016/j.mce.2010.01.001 20060878

[B29] Voth H Oberthuer A Simon T Kahlert Y Berthold F Fischer M Co-regulated expression of HAND2 and DEIN by a bidirectional promoter with asymmetrical activity in neuroblastoma BMC Mol Biol 2009 10 28 10.1186/1471-2199-10-28 19348682 PMC2670301

[B30] Zanotto E Lehtonen V Jacobs HT Modulation of Mrps12/Sarsm promoter activity in response to mitochondrial stress Biochim Biophys Acta 2008 1783 12 2352 2362 10.1016/j.bbamcr.2008.08.001 18755224

[B31] Grubenmann CE Frank CG Kjaergaard S Berger EG Aebi M Hennet T ALG12 mannosyltransferase defect in congenital disorder of glycosylation type lg Hum Mol Genet 2002 11 19 2331 2339 10.1093/hmg/11.19.2331 12217961

[B32] Chantret I Dupré T Delenda C Bucher S Dancourt J Barnier A Charollais A Heron D Bader-Meunier B Danos O Seta N Durand G Oriol R Codogno P Moore SE Congenital disorders of glycosylation type Ig is defined by a deficiency in dolichyl-P-mannose:Man7GlcNAc2-PP-dolichyl mannosyltransferase J Biol Chem 2002 277 28 25815 25822 10.1074/jbc.M203285200 11983712

[B33] Robinson SW Morris CD Goldmuntz E Reller MD Jones MA Steiner RD Maslen CL Missense mutations in CRELD1 are associated with cardiac atrioventricular septal defects Am J Hum Genet 2003 72 4 1047 1052 10.1086/374319 12632326 PMC1180336

[B34] Rupp PA Fouad GT Egelston CA Reifsteck CA Olson SB Knosp WM Glanville RW Thornburg KL Robinson SW Maslen CL Identification, genomic organization and mRNA expression of CRELD1, the founding member of a unique family of matricellular proteins Gene 2000 293 1-2 47 57 10.1016/S0378-1119(02)00696-0 12137942

[B35] Maslen CL Babcock D Redig JK Kapeli K Akkari YM Olson SB CRELD2: gene mapping, alternate splicing, and comparative genomic identification of the promoter region Gene 2006 382 111 120 10.1016/j.gene.2006.06.016 16919896

[B36] Ortiz JA Castillo M del Toro ED Mulet J Gerber S Valor LM Sala S Sala F Gutiérrez LM Criado M The cysteine-rich with EGF-like domains 2 (CRELD2) protein interacts with the large cytoplasmic domain of human neuronal nicotinic acetylcholine receptor α4 and β2 subunits J Neurochem 2005 95 6 1585 1596 10.1111/j.1471-4159.2005.03473.x 16238698

[B37] Nundlall S Rajpar MH Bell PA Clowes C Zeeff LA Gardner B Thornton DJ Boot-Handford RP Briggs MD An unfolded protein response is the initial cellular response to the expression of mutant matrilin-3 in a mouse model of multiple epiphyseal dysplasia Cell Stress Chaperones 2010 15 6 835 849 10.1007/s12192-010-0193-y 20428984 PMC3024081

